# Facilitating admissions of diverse students: A six-point, evidence-informed framework for pipeline and program development

**DOI:** 10.1007/s40037-017-0341-5

**Published:** 2017-02-28

**Authors:** Meredith E. Young, Aliki Thomas, Lara Varpio, Saleem I. Razack, Mark D. Hanson, Steve Slade, Katharine L. Dayem, David J. McKnight

**Affiliations:** 1 Department of Medicine, Faculty of Medicine, McGill University, Montreal, Quebec Canada; 2 Centre for Medical Education, Faculty of Medicine, McGill University, Montreal, Quebec Canada; 3 Department of Physical and Occupational Therapy, Faculty of Medicine, McGill University, Montreal, Quebec Canada; 4 Department of Medicine, Uniformed Services University of the Health Sciences, Bethesda, MD USA; 5 Department of Pediatrics, Faculty of Medicine, McGill University, Montreal, Quebec Canada; 6 Department of Psychiatry Faculty of Medicine, University of Toronto, Toronto, Ontario Canada; 7 Royal College of Physicians and Surgeons of Canada, Ottawa, Ontario Canada; 8 Department of Anesthesia, Faculty of Medicine, University of Toronto, Toronto, Ontario Canada

**Keywords:** Diversity, Medical education, Knowledge translation, Admissions

## Abstract

**Introduction:**

Several national level calls have encouraged reconsideration of diversity issues in medical education. Particular interest has been placed on admissions, as decisions made here shape the nature of the future physician workforce. Critical analysis of current practices paired with evidence-informed policies may counter some of the barriers impeding access for underrepresented groups.

**Methods:**

We present a framework for diversity-related program development and evaluation grounded within a knowledge translation framework, and supported by the initiation of longitudinal collection of diversity-related data. We provide an illustrative case study for each component of the framework. Descriptive analyses are presented of pre/post intervention diversity metrics if applicable and available.

**Results:**

The framework’s focal points are: 1) data-driven identification of underrepresented groups, 2) pipeline development and targeted recruitment, 3) ensuring an inclusive process, 4) ensuring inclusive assessment, 5) ensuring inclusive selection, and 6) iterative use of diversity-related data*. *Case studies ranged from wording changes on admissions websites to the establishment of educational and administrative offices addressing needs of underrepresented populations.

**Conclusions:**

We propose that diversity-related data must be collected on a variety of markers, developed in partnership with stakeholders who are most likely to facilitate implementation of best practices and new policies. These data can facilitate the design, implementation, and evaluation of evidence-informed diversity initiatives and provide a structure for continued investigation into ‘interventions’ supporting diversity-related initiatives.

## What this paper adds

Increasing focus has been placed on the diversity of the future physician workforce, and role of admissions in shaping that workforce. This paper provides a framework for organizing pipeline and program development with a focus on maintaining and expanding the diversity of medical students. Each component of the framework is paired with a case study of changes intended to support diversity, ranging from establishing new Offices to subtle changes in websites. This paper reports on an iterative and supportive collaboration between research, data collection, and knowledge translation with the goal of identifying diversity targets, and supporting the implementation of evidence-informed initiatives to support an inclusive admissions process.

## Introduction

The need for social accountability and for diversification within the medical student population is widely acknowledged by the academic medicine community [1–3]. Empirical research has reported the underrepresentation of certain population groups among medical school matriculants relative to regional and national metrics across several international contexts [4–6]. While the specific underrepresented population varies by regional, national, and international context, individuals from lower socioeconomic backgrounds are frequently underrepresented [5–9]. Several national-level policies call upon educational institutions to attend more carefully to issues of diversity in health professions education [10, 11]. Scholars suggest that expanding the diversity of health care professionals can support culturally competent care and facilitate access to care for traditionally underserved populations (e. g. [12]).

These calls to action resonate across the health professions education community; they are particularly relevant to medical school admissions committees whose decisions determine the composition of the future physician workforce. Indeed, greater diversity in the physician workforce must begin with medical school admissions practices [10]. The development, implementation, and evaluation of medical school admissions practices must address the needs and concerns of the many stakeholders involved in admissions policies – at both local and national levels. These practices should also be grounded in effective processes for moving diversity-related evidence into admission practices and policies.

In this paper, we present the diversity-related admissions program developments designed, implemented, and evaluated at two Canadian medical schools participating in our research collaboration [9]. These developments were grounded in empirical evidence, responded to local diversity-related contextual considerations, and were informed by the knowledge translation process and literature. The purpose of this report is to consolidate the successful diversity-related efforts into a six-point framework for pipeline and program development aimed at improving the diversity of medical school matriculant populations. We first discuss the knowledge translation framework that informed our efforts and the diversity-related research and quality assurance findings that grounded design and initial evaluation of our programs. We then describe each point of the developed six-point framework in relation to a case study to illustrate the suggested program component, along with outcome data where available.

### Conceptual framework for generating and implementing diversity evidence: knowledge translation

Knowledge translation provides the framework for moving admissions-relevant diversity-related evidence into practice. The Canadian Institute of Health Research defines knowledge translation as ‘a dynamic and iterative process that includes synthesis, dissemination, exchange and ethically sound application of knowledge. This process takes place within a complex system of interactions between researchers and knowledge users that may vary in intensity, complexity and level of engagement depending on the nature of the research and the findings as well as the needs of the particular knowledge user’ [13].

The two major knowledge translation concepts relevant to our research are 1) the creation (generation) of knowledge, reflected in the creation of diversity-related metrics and data for matriculants and 2) the application of evidence, namely the application of the findings from monitoring locally relevant diversity data to admissions processes. Knowledge translation emphasizes aligning evidence with the needs of knowledge users in a specific local context (i. e. admissions and undergraduate medicine programs in our participating schools). Knowledge translation is predicated on the notion that, in order for evidence or new knowledge to inform or improve practice, it must be relevant, timely and useful for the intended knowledge users [14, 15]. We draw from the ‘knowledge-to-action’ cycle, which provides a holistic view of the knowledge translation phenomenon by emphasizing the fluid boundaries between ‘knowledge creation’ and ‘action’ [15, 16], to guide our reflections on how we can move diversity research evidence into admissions best practices [14]. The knowledge-to-action framework privileges social interaction amongst relevant stakeholders and adaptation of research evidence that takes local context and culture into account [14–16].

As we progressed in our work and examined how diversity-related data could be used to inform admissions practices, we considered the seven major action stages of the knowledge-to-action framework, i. e. identifying a problem in the practice/knowledge gap; identifying and selecting the knowledge to be implemented to address the gap; adapting or customizing the knowledge to the local context; evaluating the determinants of the knowledge use; selecting, tailoring, and implementing interventions to address the knowledge/practice gap; monitoring the knowledge use in practice; evaluating the outcomes or impact of using the new knowledge; and determining strategies for ensuring that the new knowledge is sustained [14]. In particular, we discussed the nature of the available diversity evidence, the need to move this evidence into practice, and how this evidence could be customized to each local context. We consulted and engaged stakeholders at local sites to proactively identify potential barriers and facilitators to implementing the knowledge. While the following descriptions of the six-point framework for pipeline and program development focus on the evidence-to-practice-via-customization action stages, we are continuously monitoring these knowledge use efforts. Over the past five years, we have been conducting ongoing evaluations of the outcomes or impacts of using the new knowledge (findings from monitoring diversity-related data) and are determining strategies for ensuring sustainability of these knowledge translation initiatives [14].

### Diversity and admissions evidence

Multiple efforts aimed at improving the diversity represented in medical schools’ applicant and matriculant populations have been undertaken at many institutions. Examples include pipeline programs and targeted recruitment initiatives [17], specialized programs with separate selection criteria [18], programmatic efforts targeted at supporting particular populations [19, 20], and continued critical analysis of selection practices and institutional contexts [21–23]. While these initiatives have reported encouraging findings, few studies have been able to report on the efficacy of diversity-related efforts due to the institution-specific nature of programs and the typically small numbers of students and applicants (with the notable exception of some large post baccalaureate programs) [24]. This may limit the applicability of traditional best-evidence approaches for pipeline and diversity-related program development and evaluation [25]. Specifically, aspects such as generalizability across sites and large enough sample sizes for traditional statistical analyses may be both unattainable and inappropriate in diversity-related initiatives. This is problematic, as the knowledge translation literature and frameworks explicitly highlight the need for local context and culture considerations to shape diversity initiatives appropriately.

As a result of this need, the development and implementation of diversity-oriented programs requires the collection and maintenance of longitudinal diversity-related data. These data, in turn, rely on the dimensions of diversity that an institution deems noteworthy and deserving of tracking. In a previous publication, our team called for a broader conceptualization of diversity [9], leading us to develop and encourage the adoption of a nationwide database that reflects locally determined diversity-related metrics while allowing medical educators to compare their local metrics against other aggregate data, be they local, regional, national or international.

### Moving knowledge into practice

As an essential component of ‘knowledge creation’, our team has been tracking surface and deep markers of diversity among medical school matriculants across five medical schools in Canada over the last five years alongside the monitoring of similar diversity metrics in our applicant pools. Our team of collaborators meets monthly to discuss: 1) data collection and analysis [14]; 2) pipeline and other diversity-related programs that have been developed and implemented individually at each site; and 3) relevant publications and evidence of the successes and obstacles affecting the diversity of our medical trainee populations.

## Methods

### Overall design

We conducted a descriptive, exploratory, multiple case study with six cases (units of analysis: individual strategies for diversity program development) [26]. These cases exemplify how locally collected and locally relevant diversity data are translated into practice to facilitate diversity-related admissions program development. Data used for this analysis include those collected using a locally developed diversity survey and local quality assurance program evaluation. Analyses include descriptive analyses of demographic data, substantiated by a report of the diversity-related admissions program details.

### Data sources

Locally relevant diversity-related data used to support the institution of the initiatives described in each case were drawn from the Health Professions Student Diversity Survey (HPSDS) [9], the Health Professions Applicant Diversity Survey (HPADS) [27] and/or local quality assurance program evaluation data at the participating institutions (McGill University and the University of Toronto, the two institutions that have been collecting data via the Health Professions Student Diversity Survey for the longest period of time, which facilitates longitudinal tracking of intervention success). Each participating institution used the Health Professions Student Diversity Survey and/or the Health Professions Applicant Diversity Survey within their local context. Data were used internally to support and evaluate most of the initiatives described below. Data relating to medical student diversity, combined across all institutions participating in the Health Professions Student Diversity Survey, can be found elsewhere [9].

### Framework development

The six-point framework presented in this paper was initially developed based on the collective experience of the team, and iteratively derived and clarified through team meetings, consensus building on the key framework components, and consultations with local stakeholders. The framework was further refined through the close examination of the individual case studies within the research team.

### Data analysis

Data analysis was descriptive including reporting frequencies, proportions, and percentages as appropriate.

## Results

Each focal point of our framework for diversity-related program development is described, first in abstract terms, and is then illustrated with a case study from one of our participant institutions. Consistent with a key knowledge translation concept (i. e. tailoring knowledge to a particular context facilitates its sustained use), our case study descriptions demonstrate how locally relevant diversity data can translate into the development, sustainability, and evaluation of a diversity-related admissions program [14, 28, 29]. When the number of participant responses was less than 5, raw numbers were not reported in order to protect the identity of the students or applicants.

### Study context

Collecting demographic data among student populations in Canada remains controversial. Consequently, the collection and analysis of demographic and diversity data for medical school applicants, medical students, residents, and practising physicians in Canada have been relatively recent and require voluntary disclosure of diversity information by applicants or registered medical students. Thus, findings such as the discordance between medical classes and the general population with respect to diversity have been a largely newly documented phenomenon in Canada [7–9].

## Framework for developing diversity-related admissions best practices

### 1) Data-driven identification of underrepresented groups

The first focal point in our framework is designed to encourage demographic data collection that in turn can enable evidence-based program development. This first step is consistent with the knowledge translation framework of starting initiatives by identifying current practices and best practice gaps. We posit that processes and tools for monitoring medical student diversity markers and the identification of locally relevant underrepresented groups must be developed and employed. Given the specificities of each medical school and their contexts (e. g. local patterns of immigration and hence underrepresentation), the collection and interpretation of findings within the local context is essential [30].

#### Case study

Our team has developed and used a tool (the Health Professions Student Diversity Survey, HPSDS [9]) to collect diversity-related data at each participating medical school in or﻿der to identify underrepresented groups. The data collected via the Health Professions Student Diversity Survey has allowed hypothesizing of the barriers impeding these groups at both the application and the admission levels. To illustrate, the Health Professions Student Diversity Survey data collected between 2009 and 2011 revealed a marked underrepresentation of Filipino Canadians in medical classes [9]. Upon consideration of specific barriers and diversity intersections that may be contributing to this observation [9], we noted the unique pattern of immigration for persons from the Philippines to Canada – a heavy weighting towards immigration based upon a personal caregiver program rather than unrestricted immigration [31]. While the significance of this observation is not yet clear, it does al﻿low us to hypothesize on how Filipino underrepresentation might be addressed specifically in outreach or pipeline programs. This finding attests to the added value of an explicit method for collecting diversity data, such as the Health Professions Student Diversity Survey, as Filipino student underrepresentation within our medical schools was not part of Canada’s historical diversity medical student discourse. It is important to note that the data-driven identification of underrepresented groups is not intended to support a parity-focused approach to admissions, but rather to assist in the identification of underrepresented groups, which is a crucial precursor to understanding the barriers to medical school admission.

### 2) Pipeline development and targeted recruitment

Once data are being routinely collected about the demographics of medical school applicants and matriculants, they can be compared with national and local population demographics to inform pipeline and recruitment efforts. When underrepresentation(s) in the applicant and matriculant pools are identified, focused analysis should be conducted into the potential structural barriers in the application and/or admissions processes that impede access for the identified population [32, 33]. In line with the knowledge translation framework, individual medical schools can implement this strategy as a best practice for supporting a more diverse student population [34].

Within our current framework, we consider a targeted recruitment initiative as a distinct component of a pipeline program. A pipeline program involves some commitment to the underrepresented student population of interest beyond a ‘one-off’ recruitment session. It typically involves multiple different interventions, in which students’ participation is tracked, encouraged, and nurtured. It also entails a commitment to sustainable community engagement, with the active seeking of design and delivery input from the community that it aims to serve and a conscious cultivation of relationships with community institutions such as schools and government [35].

#### Case study

The Quebec First Nations and Inuit – Faculties of Medicine program [36], which reserves four places annually for qualified Indigenous students in the four faculties of medicine in Quebec, resulted from a formal negotiation and discussion between the faculties of medicine in Quebec, the Council of Band Leaders of Quebec and Labrador, and the Government of Quebec. Within this program, one university participating in our collaboration has six individuals, who self-identify as Indigenous, enrolled at various stages of training (between 2008 and present) [37] (compared with four individuals who self-declare as Indigenous being awarded medical degrees between 1821 and 2007) [38]. This program is considered comprehensive in that it contains pipeline outreach, more proximal interview preparation sessions, joint selection with both the medical schools and members of Indigenous communities, and Indigenous student support while in medical school.

### 3) Ensuring an inclusive process

Ensuring an inclusive process requires conscious attention to 1) supporting and being welcoming to persons from underrepresented groups and 2) institutional measures to maximize the inclusivity of admissions-related processes. This has the potential for broadening the pool of applicants and the pool of admitted students to a medical school. This is an essential component of the knowledge translation process; once the underrepresented populations have been identified, Faculties must continue to monitor the nature of the process, its sustainability, and its success in identifying these populations and potential barriers.

#### Case study

There are no Canadian data regarding self-reported sexual orientation rates among the population age group that characterizes medical school applicants. However, the United States Census Bureau National Health and Social Life Survey estimates as many as 8% of individuals 18 to 34 years of age self-reported homosexuality or bisexuality [39]. In the first year that the Health Professions Student Diversity Survey was used in one of our local contexts the rate of reporting non-heterosexual orientations was only 2.3% of registered medical students at this institution. The local medical school team considered two possible explanations for this: 1) that the medical student body was not diverse in terms of sexual orientation or 2) that the student body was diverse, but the medical school environment was not one in which they were comfortable reporting non-uniquely heterosexual orientations. Therefore, an explicit ‘non-discrimination and welcoming of applications from diverse groups’ statement was placed on the admissions website. Since the inclusion of this statement, rates of reporting non-heterosexual orientations have increased (4.1 to 8.2% in the subsequent three years). It remains unclear as to whether this represents an increase in diversity or an increase in reporting; however, the data seem to support the value of small diversity-related changes to increase and manifest admissions inclusiveness.

### 4) Ensuring inclusive assessment

This strategy focal point requires the examination of traditional applicant and admissions assessment methods to identify barriers that may contribute to underrepresentation of certain groups. This strategy is akin to a continuous monitoring of the ‘best practice’. Central to the knowledge translation process is the notion that all stakeholders must ensure that the evidence is up to date and that it remains relevant and useful for the programs.

#### Case study

One participant school in 2011–2012 implemented a strategy to manage perceived, potential, and actual conflicts of interest within the admissions assessment process. The conflict of interest strategy restricts both faculty and student raters (those involved in file review and interviews) from providing admissions advice that is not publically available knowledge (‘insider knowledge’) to current or anticipated applicants. This initiative arose from the observation that a large percentage of registered medical students had a physician parent [7]. This high proportion of individuals with a physician family member raises concerns regarding potential conflicts of interest between candidates and potential assessors. Admissions committee members and all faculty and student raters must explicitly declare their potential conflicts in relation to their ability to serve as an assessor in the admissions process, according to stated definitions (for example, knowing a relative who is applying to that medical school in the current admissions cycle). Rater-declared potential conflicts (such as knowing or being related to someone currently applying to medical school) are reviewed by an adjudication panel before raters may participate in file reviews or interviews. Outcomes from this adjudication panel include: able to participate as raters, able to participate as raters but are ‘isolated’ from those applicants for whom they may have the potential conflict, and unable to participate as raters for the current admissions cycle.

This strategy remains in effect, and in the 2014/2015 admissions cycle, 596 individuals registered to participate as admissions raters. The adjudication panel reviewed 97 rater self-declarations of potential conflicts of interest (16% of total admissions raters). The panel advised 25% (24/97) of these raters that they could not participate in the current admissions cycle and 75% (73/97) that they could participate but were restricted to review files and conduct interviews for individuals for which they had no potential conflict and were not to discuss their admissions ‘insider knowledge’ with those applicants identified within conflict of interest declarations [40].

### 5) Ensuring inclusive selection

This focal point calls for the investigation of the underrepresentation of applicants from specific populations (e. g. lower socioeconomic backgrounds), and of the barriers within the admissions processes that may be contributing to this. Again, this demonstrates the need to continually monitor best practice within a local context and ensure stakeholders’ needs are being met.

#### Case study

At one participant school, a Faculty diversity statement was adopted in 2011 identifying three student groups as underrepresented in medical education, for priority attention: 1) Indigenous Peoples of Canada (First Nations, Inuit, and Métis), 2) people of African ancestry, and 3) the economically disadvantaged. Making the Indigenous Peoples of Canada student group a priority for attention necessitated programmatic review of a range of strategies focused upon admissions, curriculum, and student support. Newly implemented strategies include an Indigenous Student Application Program (ISAP), a culturally safe admissions process for Indigenous student applicants, and an Office of Indigenous Medical Education with two new Faculty Leads in Indigenous Health Education, and an Indigenous Health Program Coordinator. Data from the Health Professions Student Diversity Survey and from the Admissions Office indicate that since the inception of the Indigenous Student Application Program there is growing Indigenous medical student representation across all medical school years (the actual numbers of students per year remain small and are not reported to protect identity of students) and the Indigenous student applicant pool has on average doubled [40]. The Office of Indigenous Medical Education also provides an educational platform for further integration of issues of Indigenous health within this participant school’s curriculum.

### 6) Iterative use of diversity-related data

The continued and iterative use of longitudinal diversity-related data to enhance and continually monitor diversity programs and pipeline developments is critical, as when new knowledge is generated and practices change, so do the strategies that are used to inform and improve future practices.

#### Case study

Collecting objective data relating to a wide range of diversity dimensions [9] has facilitated, supported, and provided the initial supportive evidence for the programs described above. Despite the relatively objective data founding these initiatives, we acknowledge that diversity data can be interpreted in politically charged and contentious ways. Independent oversight of diversity initiatives may be warranted. One participant institution developed a Widening Participation Committee in order to manage, interpret, and act upon diversity-related data. As a Faculty committee with medical, interprofessional, and community representation, it is completely separate from the student selection process and produces an annual report with identified diversity activities for the coming year based upon interpretation of student demographic surveys (i. e. the Health Professions Student Diversity Survey [9]). This committee represents an attempt to formalize the enactment of social accountability with respect to representative diversity in medical school, but it remains an independent advisory body in order to act as ballast to the range of possible interpretations of diversity-related data.

## Discussion

The six-point framework for pipeline and program development we propose encourages targeted evidence-informed strategies both ‘small’ (i. e. language changes on Admissions Office webpage) and ‘large’ (i. e. the creation of Offices to support particular underrepresented populations), for program development. While these interventions and programs are not entirely novel, the explicit linking of these programs to comprehensive local databases that include a wide range of dimensions of diversity is a novel contribution. The data presented here relied on the Health Professions Student Diversity Survey [9] and local quality assurance programming, but these are simply the tools that provided data on which these programs grew.

Developing coordinated data-basing initiatives containing diversity-focused metrics at the regional, national, and perhaps international level would allow the identification of gaps in representation amongst registered medical students and applicants, fostering the development and evaluation of diversity programs. The identification of locally relevant metrics, the collection and iterative use of locally relevant data, and associated program development is key in ensuring integration and sustainability of best practices, permits a means for quality assurance of medical admissions processes, and can help support external review of such programs; in particular to accreditation processes [41].

The data generated by the Health Professions Student Diversity Survey and local quality assurance evaluation are a demonstration of how locally relevant primary and evaluative evidence can support evidence-informed policy making [25, 42]. This is consistent with a central tenet of knowledge translation as various sources of information, including knowledge from research findings, quality assurance evaluation, and different forms of knowing such as experiential knowledge, are considered ‘legitimate’ sources of knowledge [14]. We hope that the focal points in our framework can provide a taxonomy to facilitate comprehensive attention to different levels of potential ‘intervention’ to support diversity-related initiatives, and open conversations regarding supporting diversity through locally grounded evidence-informed approaches.

We suggest that diversity-related admissions practices (both the development of novel approaches and critical evaluation of accepted practices) can function as an area for engaged scholarship, a concept that is currently at the heart of much of the knowledge translation discourse, and is defined as a form of collaborative inquiry between academics and practitioners that leverages differences in perspective in order to generate knowledge [25]. It is based on the notion that true collaboration and integration of diverse perspectives from multiple stakeholders can result in the uptake of high-quality and relevant research findings. This collaborative approach is expected to optimize the likelihood that evidence will be used to change behaviours and improve practice [43, 44].

Through our analysis, and grounded in the principles of knowledge translation, we hope to emphasize the following points:

1. Diversity-related data must be collected on a wide range of markers identified through collaboration with multiple stakeholders [9];
2. Potential barriers to implementing data collection tools, and possible solutions, must be considered at the outset of program development;
3. A local champion should be identified to shepherd the implementation and maintenance of data collection processes;
4. Continuous and ongoing discussion and monitoring of implementation efforts must be supported through engaged scholarship [25];
5. Data should inform the development and evaluation of pipeline and program initiatives;
6. Assessment processes must be built into the pipeline and program initiatives; and
7. As the knowledge translation literature suggests, it is necessary to ‘begin with the end in mind’ [14, 45, 46]. Diversity-oriented program efforts must consider what knowledge is generated, who the targeted end-users are, and build in processes to support stakeholders in embracing and applying the knowledge.

The collaborative collection of longitudinal and multidimensional diversity data and continued research and quality assurance evaluation of program development is essential for medical schools to systematically meet the healthcare needs of the nation’s diverse population through ensuring a diverse physician workforce.
